# Using an Active Screwed Nasoalveolar Molding Device for Defect Rehabilitation in Patients With Bilateral Cleft Lip and Palate

**DOI:** 10.7759/cureus.68204

**Published:** 2024-08-30

**Authors:** Aida M Mossaad, Moustapha A Abdelrahman, Salem A Waly, Ahmed M Sapri, Wael Ghanem, Shadia Abdelhameed N Elsayed

**Affiliations:** 1 Oral Surgery, National Research Center, Cairo, EGY; 2 Orthodontics, National Research Center, Cairo, EGY; 3 Oral and Maxillofacial Surgery, Al-Azhar University, Cairo, EGY; 4 Oral and Maxillofacial Surgery, Mansoura University, Mansoura, EGY; 5 Pediatric Plastic Surgery, Ain Shams University, Cairo, EGY; 6 Oral Surgery, Al-Azhar University, Cairo, EGY

**Keywords:** nasal device, lip., cleft, molding, naso alveolar

## Abstract

The present case series aimed to assess non-surgical elongation of the columella and reduction of cleft gaps in patients with bilateral cleft lip and palate using the active nasoalveolar molding (ANAM) device and tissue expansion principles. The study included six complete bilateral cleft patients aged one month: three males and three females. A nasoalveolar molding technique was applied using an active device (ANAM) with a 3D screw, worn by infants for two months. The activation protocol for screw closure is approximately 0.25 mm (quarter turn) on alternating days, resulting in almost 1 mm per week and 4 mm per month and reaching 8 mm after eight weeks. Evaluation involved measuring lip defect sizes and the nostril gap, columellar length and rotation of premaxilla before and two months after the ANAM period before surgical repair. The results show that the anterior rotation of the premaxilla and the lip and nostril gaps were significantly reduced (*p *< 0.05), with maximum reduction in the anterior rotation of the premaxilla (mean difference ± SD was 4.22 ± 0.4). Simultaneously, the columellar height was significantly increased with a mean difference ± SD of 2.0 ± 0.4 (*p* < 0.001). The current case series demonstrated that the ANAM device is a safe and effective technique for decreasing the lip and nostril gaps, repositioning the protruded premaxilla, and elevating the depressed columella. No side effects were recorded in current cases.

## Introduction

Nasoalveolar molding (NAM) is a customized orthodontic technique used for molding the nasoalveolar maxillary arch in infants suffering from unilateral or bilateral cleft lip and palate [1,2]. It facilitates infant feeding by sealing the defect and acts as a presurgical therapy that minimizes the width of the cleft gap over several months preoperatively, facilitating lip repair surgery for aesthetic and functional outcomes [3-5].

The complexity of bilateral cleft lip and palate (BCLP) patients usually results from the protruded rotated premaxilla with significantly increased alar base width, depressed naris, flattened nasal tip with deficient or absent columella, and widely separated lip segments. More extensive wide clefts are typically associated with more severe nasolabial deformities [6,7]. Therefore, these cases require a multidisciplinary approach involving multiple surgical interventions and long-term orthodontic and prosthetic management [6].

Recent trends now involve using virtual planning; intraoral scanners for digital impressions, which are more precise and less complex than manual ones; design simulation; 3D printing and growth projections; and artificial intelligence technology to facilitate the long surgical journey for cleft patients and parents. However, it is an expensive procedure and requires special skills [1,8-10].

Orthopedics appliances have shown varying degrees of success in terms of aligning and stabilizing the maxillary segments and improving nasal symmetry. However, active devices with active screws are superior to passive devices that only decrease the size of the cleft defect and minimize the lip and nostril gaps. Moreover, active devices have shown improved clinical outcomes, with a larger role in repositioning the protruded maxilla with backward rotation, as well as increasing the columellar height [1,8-11].

In Egypt and other countries, BCLP conditions present a considerable burden, both psychologically for the patient and financially for the healthcare system. The present study hypothesized that using an active screwed nasoalveolar molding (ANAM) device for defect rehabilitation in patients with BCLP improves clinical outcomes by effectively reshaping the alveolar ridges, nasal cartilages, premaxilla, and lips, facilitating subsequent surgical procedures and enhancing overall aesthetic and functional results.

Therefore, this study aimed to evaluate the effectiveness of the ANAM device in the rehabilitation of BCLP in Egyptian patients. The study sought to establish whether this innovative approach offers superior results in terms of functional and aesthetic outcomes, ultimately improving the quality of life for patients with BCLP.

## Materials and methods

Study design and setting

This case series study included eight complete bilateral cleft patients aged one month: three males and three females. A NAM technique was applied with the ANAM device with a 3D screw, worn by infants for two months. The activation protocol for screw closure is approximately 0.25 mm (quarter turn) on alternate days, equating to almost 1 mm per week and 4 mm per month, reaching 8 mm after the infant has worn it for eight weeks. Evaluation involved measuring the size of lip defects, the nostril gap, rotation of premaxilla and columellar length before and two months after the ANAM period before surgical repair. The current study was approved by the Medical Ethical Research Committee of the National Research Center Egypt and adhered to the Helsinki Declaration on Clinical Research Principles (MERC 19255/2023). Patients’ guardians signed an informed consent form and were informed about the study’s purpose.

Impression-taking

**Figure 1:**
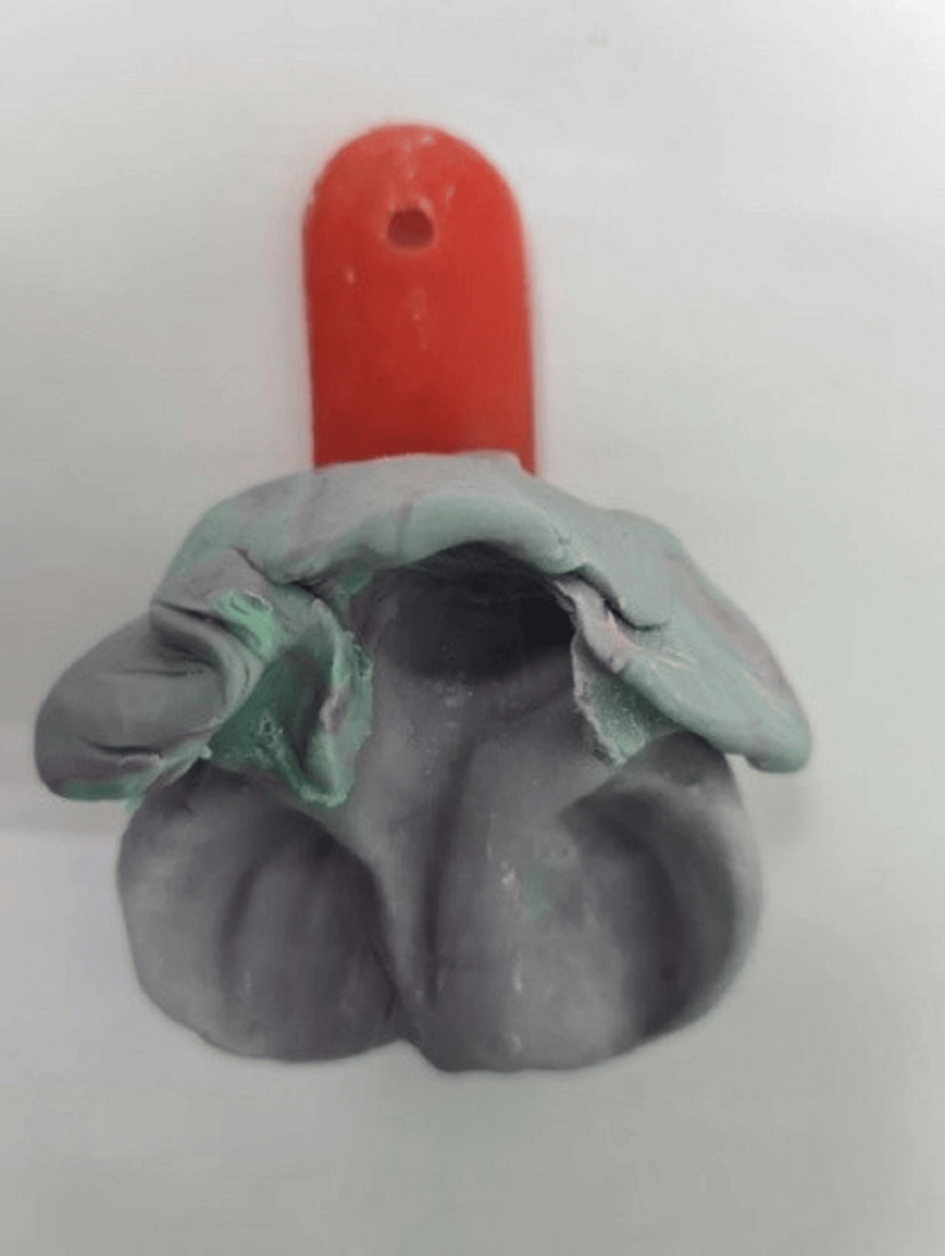
Photograph showing silicone impression in a custom tray

It was suggested to parents that they should wait at least three to four hours before taking the baby’s impression. This waiting period helps avoid aspiration and ensures the baby's safety. The baby was held at 45 degrees. A special tray for pediatrics was chosen. An impression was taken with silicone material without obstructing the airway. Dental stone was poured into the impression to create a mold (Figure 1).

ANAM device fabrication

The undercuts on the cast were blocked, and a separating medium was added. The plate on the cast was fabricated from self-cure acrylic resin. The plate should be at least 2 mm thick to ensure rigidity. The stent is made from 0.3-inch nickel-chromium alloy. The plate was placed in the mouth, and emerging wires were bent to the nasal part via a stent. After checking the adaptation and stability of the device, the soft acrylic part was placed in the medial alar in the nasal part of the cleft side.

Device application

**Figure 2:**
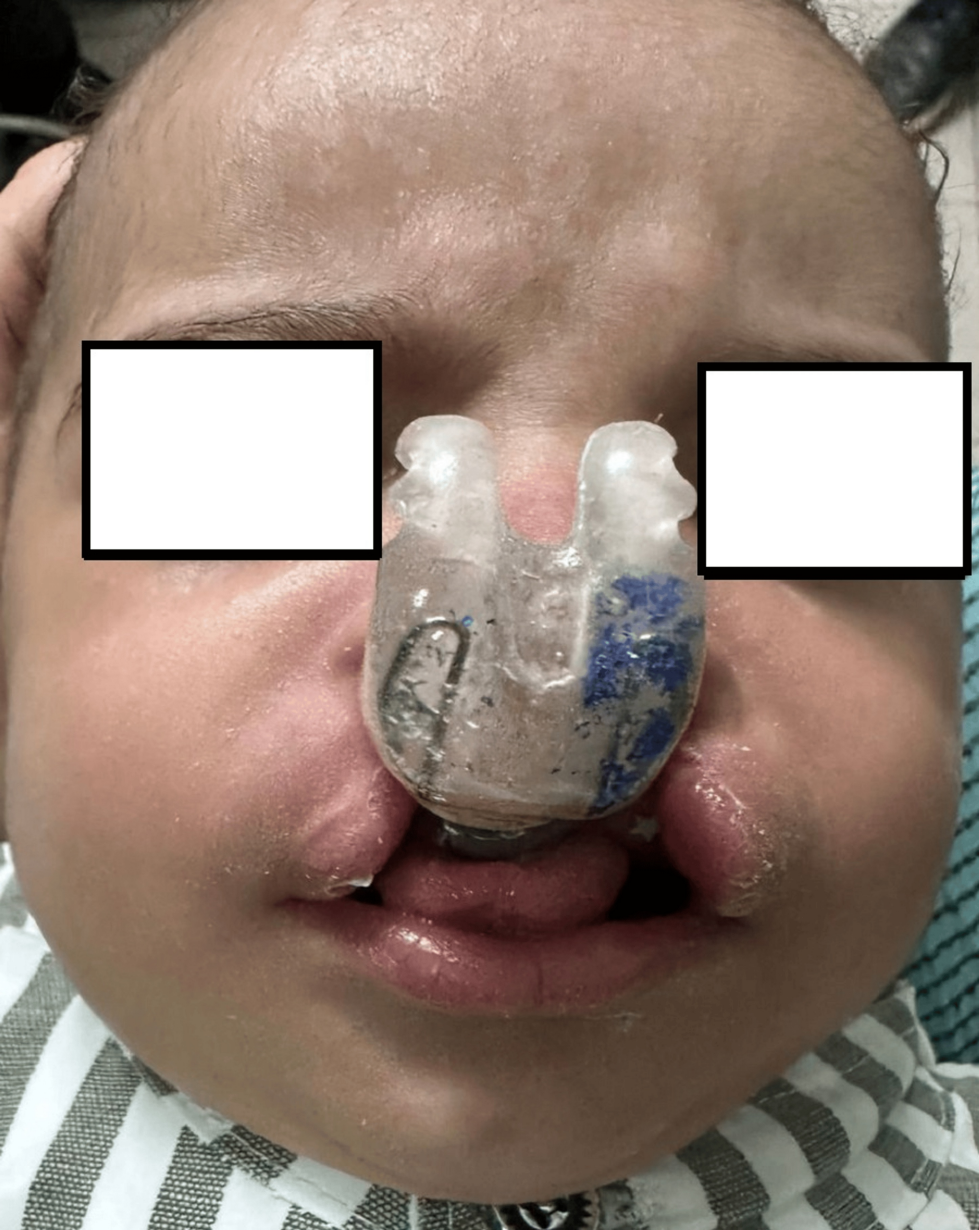
Photograph showing the active nasoalveolar molding (ANAM) device in place.

The device should be placed carefully and fixed with surgical strips. The nasal airway should be free from obstructions, and the molding plate should seal the palatal defect. The tape should be placed at the base of the nose (nasolabial angle) rather than low on the lip at the vermilion border (Figure 2).

Follow-up and data collection

Weekly visits were scheduled to check the device adaptation; the molding plate should be properly relieved to avoid exerting excessive pressure causing inflammation or ulceration of soft tissue. Instructions for applying and removing the surgical tape are given to the parents, indicating gentle removal to avoid skin irritation. An evaluation was performed by clinical observation and taking photos. A caliper ruler was used to measure the size of defects, lip gap (distance from lip to premaxilla), nostril gap (distance from lateral nasal wall to premaxilla), and columellar height (distance from inferior columellar base to upper nasal point) and evaluate premaxillary rotation: anterior rotation of the premaxilla before and after the ANAM period using a caliper ruler after eight weeks.

Statistical analysis

All data was processed and analyzed using SPSS version 25.0.0 (IBM Corp., Armonk, NY, USA). Preoperative and postoperative measures were compared. The mean difference after the intervention was compared using the paired t-test. The difference was considered statistically significant when p < 0.05 and highly statistically significant when p < 0.01.

## Results

The present study started with eight cases. Two cases refused to delay the cleft repair surgeries; they did not undergo the ANAM process and were excluded from the study. Consequently, this study included six cases in which the patients agreed to cooperate with our cleft clinic. Weekly follow-up visits were scheduled to monitor the progress and commitment to wearing the device. Clinical observation showed the adaptation of infants to applying the device after the first few days. The device also helped them with feeding and swallowing, as reported by their parents. It was explained to them that the delay in surgical closure, which was extended for almost two months longer than usual, would obtain better aesthetics and functional results and decrease the number of surgeries required later. Some difficulties and complications were reported. One case finished activating the device after only seven weeks, as the device screw stopped spontaneously. Another case faced device fracture from the acrylic part and stopped for one week in the middle of the activation period until a new device was manufactured. Two cases complained of difficulty in wearing the device and applying the tape regularly due to mucosal and skin irritation. They were advised to clean the area better with soaked cotton before applying the device. Clinical observations and photographs were compared. A comparison between pre- and two months post-ANAM, involving using a caliper ruler and recording the measurements, revealed that the anterior rotation of the premaxilla and the lip and nostril gaps were significantly reduced (p < 0.05). Maximum reduction was observed in the anterior rotation of the premaxilla (mean difference ± SD was 4.22 ± 0.4 mm), followed by the lip gap and then the nostril gap, with a statistically highly significant difference (p < 0.01). Simultaneously, the columellar height was significantly increased, with a mean difference ± SD of 2.0 ± 0.4 mm (p < 0.001), as shown in Table 1.

**Table 1:** Comparison between pre- and postoperative measurements (after two months of nasoalveolar molding)

Measurement (mm)	Pre Mean±SD	Post Mean±SD	Difference Mean±SD	P value#
Lip gap	6.63±0.5	3.67±0.6	-2.97±0.4	<0.001*
Nostril gap	4.83±0.5	2.50±0.8	-2.33±0.5	<0.001*
Columellar height	1.50±0.5	3.50±0.6	2.0±0.4	<0.001*
Anterior rotation of premaxilla	7.70±0.4	3.48±0.8	-4.22±0.4	<0.001*

**Figure 3:**
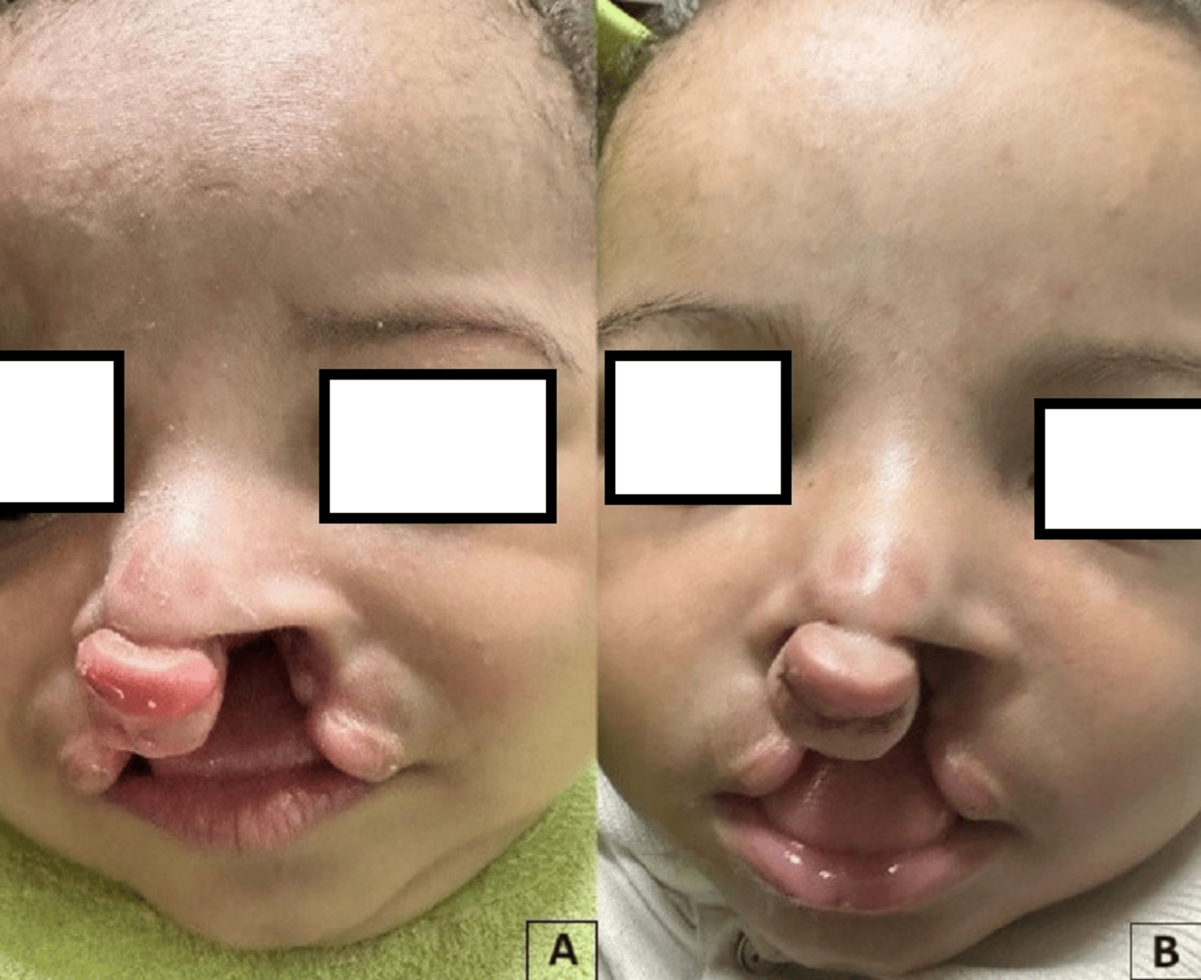
Clinical photograph showing preoperative versus postoperative active nasoalveolar molding (ANAM) application.

The weekly activation of the 3D ANAM screw device for eight consecutive weeks achieved almost 8 mm backward rotation of the premaxilla. The parents noticed the difference by the end of the treatment period and were satisfied with the outcome, observing the decreased lip gap, reduced alar base width, and non-surgical increase in the columellar height. They also mentioned that the infants increased in weight due to better feeding ability (Figure 3).

## Discussion

In the present study, we demonstrated that it is possible to elongate the columella in BCLP without surgery using tissue expansion principles. This was accomplished by applying stresses to the lip and nose and progressively expanding the nasal molding device.

The characteristics of a unilateral cleft deformity include a wide nostril base, a depressed dome, an enlarged alar rim with an oblique columella, an overhanging nostril apex, and divided lip segments at the cleft side. The deformed lower nasal cartilage is displaced laterally and inferiorly. The bilateral cleft side deformity results in a rotated premaxilla. The lip segments are widely separated. The increased alar base width with a flattened nasal tip and a deficient or absent columella presents the greatest challenge regarding reconstruction [12-14]. The impression-taking technique is crucial to the outcome: it is preferred to use a customized tray for safety rather than the traditional finger technique, which can cause choking or cyanosis in infants [15-17]. Moreover, fabricated trays with sulcus adaptation are more precise and save time, effort, and material consumption.

Traditional NAM devices depend on applying external forces and passive molding. The active screwed NAM device introduces a new approach using an adjustable, screw-based mechanism to apply controlled forces. This enables more precise and dynamic adjustment of the device to meet each patient’s needs, potentially leading to more effective and rapid molding of the alveolar ridges and nasal cartilage, improved patient compliance due to the reduced need for frequent device adjustments, enhanced presurgical outcomes, and a better foundation for the primary challenging surgical repair.

Many studies have shown that NAM devices have proven effective in presurgical cleft therapy, with the potential to improve aesthetics and achieve positive functional outcomes, including improved nasal symmetry and alveolar position in patients with clefts of the lip, alveolus, and palate [18-21]. The rapid NAM concept involves the virtual modification and manufacture of NAM devices [18]. A modified NAM device has also been developed to reduce the need for frequent patient visits, particularly for those traveling from distant places [19].

Presurgical narrowing of the cleft defect aims to improve surgical outcomes, minimizing scarring and healing complications. Grayson et al. (1999) [22] was the first to propose a presurgical ANAM technique comprising premaxillary orthopedics and nasal molding to decrease lip and nasal deformity prior to surgical repair. Various studies have explored the use of NAM in treating bilateral cleft lips. Patil and Nimbalkar-Patil (2018) [23] proposed a modified activation technique for the nasal stent of the NAM appliance, specifically for columellar lengthening. Ijaz et al. (2010) [24] introduced a self-retentive orthopedic plate with an anterior ring to retract and align the premaxilla, facilitating NAM and initial lip repair. Silveira et al. (2003) [25] developed a modified NAM appliance with an orthodontic wire and acrylic bulb for easier adjustment and improved symmetry. Suri and Tompson (2004) reported a muscle-activated maxillary orthopedic device for NAM in newborns with a unilateral cleft lip and palate that improved the alveolar position and nasal symmetry [20]. These studies collectively highlight the potential of NAM in treating bilateral cleft lips, with various modifications and techniques to enhance its effectiveness.

Ashith et al. (2022) [26] introduced the 3D Versascrew technique, not only relying on a passive device but also creating an active part that decreases the gap dimension mediolaterally as well as anteroposteriorly, realigning the premaxilla in position via backward rotation with promising results. Pang et al. (2017) [27] conducted the first prospective and rigorous analysis of 3D progressive changes in children with complete unilateral cleft lip and palate who underwent presurgical ANAM treatment prior to primary cleft lip surgery. Their study examined the effect of two-stage therapy; stage I involved observing alveolar segment adjustment after ANAM, and after 43 days at stage II, they added nasal stents and observed the effect of ANAM on the nostrils. Their study aimed to understand how rapidly the ANAM technique produces changes regarding molding the nasal cartilages and lengthening the columella, with correction of nostril height and weekly 3D analysis outcomes with non-invasive stereophotogrammetry. In the present study, we observed the action of a custom-made active device with a screw in the backward rotation of an inwardly protruded premaxilla, attempting to place it in position besides acting to minimize the lip and nostril gaps. Active devices are easier to control in action and predict better results. The active screw can achieve up to 8 mm anteroposterior action on a protruded premaxilla, assisting backward rotation, unlike passive devices that can only narrow the cleft defect gap. The impression-taking technique is crucial to the success of the treatment, so it is preferred to use a customized tray to reduce time and effort, as well as complications such as choking or cyanosis, rather than the classic finger technique described by Kalaskar et al. (2021) [8]. Moreover, Liang et al. (2018) [11] studied two groups of patients: the first group underwent lip closure without presurgical ANAM while the second group underwent presurgical ANAM before lip closure. They concluded that the group that underwent presurgical NAM experienced better results after long-term follow-up.

The ANAM device is advantageous in that it reduces the cleft size and the gap in the upper lip, lifts and narrows the nose with reduced future scarring and need for secondary bone grafting, and promotes healing without tension. Moreover, the child would need fewer surgeries later in their childhood [28,29]. It is a fast, simple procedure after taking an impression and manufacturing the device, which resembles an orthodontic retainer with nasal stents. It is worn constantly by the infant and is held in place with a skin tape. Unilateral cases usually take three to four months, while bilateral cases take up to five to six months [12,26,30]. Complications associated with the ANAM device include irritation of the oral mucosa and ulceration of gingival tissues resulting from pressure or rubbing.

Considering the socioeconomic and healthcare delivery context in Egypt, the active screwed NAM device could minimize the entire treatment plan period and number of multisurgical operations required for cleft patients, hence lowering healthcare expenses [19]. Additionally, improved aesthetic outcomes can significantly enhance patients’ quality of life and social integration. The current approach may be particularly beneficial in regions with limited access to specialized surgical and orthodontic care, as it provides an effective presurgical intervention that can be managed with fewer frequent adjustments. A future investigation comparing post-operative follow-up to cases treated with different devices should be considered. 

## Conclusions

The active NAM device is a safe, predictable, and effective technique for decreasing the lip and nostril gaps as well as repositioning the protruded premaxilla and elevating the depressed columella. No side effects were recorded in current cases. The active screw can reach up to 8 mm anteroposterior action on the protruded premaxilla, assisting backward rotation, unlike passive devices that can only narrow the cleft defect gap. The impression-taking technique is crucial to the success of the treatment.
